# A shared *HLA-DRB1* epitope in the DR beta first domain is associated with Vogt-Koyanagi-Harada syndrome in Indian patients

**Published:** 2010-03-05

**Authors:** Jean-Marie Tiercy, Sivakumar R. Rathinam, Marianne Gex-Fabry, Edoardo Baglivo

**Affiliations:** 1 National Reference Laboratory for Histocompatibility, Transplantation Immunology Unit, Geneva University Hospitals and University of Geneva, Geneva, Switzerland; 2 Aravind Eye Hospital & P.G. Institute of Ophthalmology, 1 Anna Nagar, Madurai, India; 3 Department of Psychiatry, Division of Adult Psychiatry, Geneva University Hospitals, Geneva, Switzerland; 4 Ophthalmology Department, Geneva University Hospitals, Geneva, Switzerland

## Abstract

**Purpose:**

Vogt-Koyanagi-Harada (VKH) disease and sympathetic ophthalmia (SO) are two distinct entities that share common clinical and histopathological features; however, it remains unknown whether they have a common genetic susceptibility. Several studies have shown an association of human leukocyte antigen (HLA)-DR4 with VKH disease in patients of different ethnic backgrounds. We present in this paper the *HLA-DRB1* genotyping analysis of a large cohort of VKH patients from southern India and compare these patients to patients with SO and to healthy individuals from the same geographic area.

**Methods:**

VKH patients were diagnosed according to the revised criteria of the International Committee on VKH disease. Patients with granulomatous uveitis after ocular trauma or multiple eye surgeries were diagnosed as having SO. Genomic DNA was extracted from all patients and controls. Samples were analyzed for *HLA-DRB1* alleles by reverse polymerase chain reaction (PCR) sequence-specific oligonucleotide (SSO) hybridization on microbeads, using the Luminex technology, and by PCR sequence-specific primers (SSP) typing for DRB1*04 allele determination. Strength of associations was estimated by odds ratios (OR) and 95% confidence intervals (CI) and frequencies were compared using the Fisher’s exact test.

**Results:**

*HLA-DRB1* alleles were determined in 94 VKH patients, 39 SO patients, and 112 healthy controls. HLA-DRB1*04 frequency was higher in VKH patients (20.2% versus 10.3% in controls; OR=2.2, p=0.005, pc=0.067). This association was lower than the association of HLA-DRB1*04 frequency in cohorts of patients from different origins. No significant DR4 association with SO was detected. HLA-DRB1*0405 and HLA-DRB1*0410 alleles were significantly increased in VKH patients (8.5% versus 0.9% in controls; OR=10.3, 95% CI=2.34–45.5, p<0.001). These two alleles share the epitope S57-LLEQRRAA (67–74) in the third hypervariable region of the HLA-DR molecule. None of the DRB1 alleles was significantly associated with SO.

**Conclusions:**

Based on the association of HLA-DRB1*0405 and HLA-DRB1*0410 alleles with VKH disease, we propose that the epitope S57-LLEQRRAA (67–74) in the third hypervariable region of the HLA-DRβ1 molecule is the relevant susceptibility epitope. This genetic component seems specific to VKH disease since no correlation could be identified in SO patients. The weaker association with *HLA-DR4* in this VKH patient cohort compared to VKH patients from northern India is probably related to the lower frequency of HLA-DRB1*0405 in our study group. The *HLA-DRB1* association with susceptibility to VKH syndrome seems weaker in Indian patients compared to Japanese or Hispanic patients, suggesting a different non-HLA immunogenetic background in Indian VKH patients.

## Introduction

Vogt-Koyanagi-Harada (VKH) disease is a serious ocular inflammatory syndrome characterized by the presence of bilateral panuveitis and exudative retinal detachments. Alopecia, vitiligo, poliosis, tinnitus, and meningeal symptoms may also be present according to the stage of the disease. Several criteria are needed to establish the diagnosis of VKH (e.g., bilateral uveitis, meningismus, and extra-ocular signs). The absence of a history of trauma/surgery is essential. Recently, revised diagnostic criteria were proposed by the First VKH International Workshop group, which classified the disease as complete, incomplete, and probable according to the presence of clinical signs [1]. Some studies have suggested that VKH is a T-lymphocyte-mediated disease and that specific auto-antigens may play a role in its pathogenesis [2,3].

Although VKH and sympathetic ophthalmia (SO) are two distinct entities, they share common clinical features (bilateral granulomatous uveitis) and histopathological signs (infiltration of T-lymphocytes in the choroid and preservation of the choriocapillaris and retina) [4].

Genetic specificities of the human leucocyte antigen (HLA) system have been associated with susceptibility to many autoimmune diseases. The initial discovery of the human leukocyte antigen (HLA)-DR4 association with VKH disease in Japanese patients [5] has been confirmed in several subsequent studies including Chinese [6], North American [7], Korean [3], Japanese [8,9], Italian [10], Hispanic [11], Mestizos [12,13], and strongly admixed Brazilian patients [14]. In several reports, the higher frequency of DR4 in VKH patients was related to the HLA-DRB1*0405 allele, and in three Japanese studies a significant increase in HLA-DRB1*0410 was observed [8,15,16]. In Mestizo patients, gene frequencies for HLA-DRB1*0102, HLA-DRB1*0404, HLA-DRB1*0407, and HLA-DRB1*0410 alleles were higher than in controls [13]. A recent study on 30 patients from Saudi Arabia reported a significant association of VKH with HLA-DRB1*0405 [17].

In the present article, we examined the frequency of *HLA-DRB1* alleles in a large cohort of VKH patients from southern India and compared this with patients with SO and healthy individuals from the same geographic area.

## Methods

### Study samples

We prospectively included 94 VKH patients, 39 SO patients, and 112 healthy subjects. All patients with VKH and SO were examined at the Uveitis Clinic of the Aravind Eye Hospital, Madurai, India.

Patients were diagnosed with VKH according to the revised criteria of the International Committee on VKH disease [1]. In the absence of a history of trauma/surgery and after exclusion of other uveitis causes, patients presenting with bilateral uveitis with exudative retinal detachment and a history of meningismus, CSF pleocytosis, or sensorineural deafness with integumentary changes were diagnosed as having complete VKH. Incomplete VKH was diagnosed in the presence of either signs or symptoms of central nervous system (CNS) involvement or integumentary changes. In the absence of both CNS and integumentary changes, patients were diagnosed as having probable VKH syndrome.

Patients presenting with granulomatous uveitis after ocular trauma or multiple eye surgeries were diagnosed with SO. For each patient other causes of granulomatous uveitis were excluded by an extensive work-up.

All patients had a complete eye examination, including Snellen visual acuity, slit-lamp biomicroscopy, tonometry, and dilated fundus examination. Intraocular inflammation was graded according to the International Uveitis Study Group recommendations [18]. Demographic data included gender, present age, and age at onset of VKH or SO. The present study was approved by the local Ethics Committee, and written informed consent was obtained from all participants.

### Analytical methods

For each subject a blood sample was taken and genomic DNA was extracted. All samples were analyzed for *HLA-DRB1* alleles by reverse polymerase chain reaction (PCR) sequence-specific oligonucleotide (SSO) reverse hybridization on microbead arrays (Luminex technology; Luminex Corporation, Austin, TX) after locus-specific amplification on genomic DNA samples, using LabType RSSO2B reagents (OneLambda, Ingen, Chilly Mazarin, France). This assay provided results at an intermediate level (groups of alleles), with assignment of the four-digit allele in some cases. The HLA-DRB1*04-positive samples were subtyped by PCR sequence-specific primers (PCR-SSP; Genovision, Milan Analytika AG, Magden, Switzerland). Intermediate resolution level data were expressed as *HLA-DRB1* two-digit types.

### Statistical analysis

Group comparison proceeded with the Fisher’s exact test for gender and disease severity and the Kruskal–Wallis test for age. Comparison of allele frequencies was performed with the Fisher’s exact test. The strength of the associations was estimated by odds ratios (OR) and 95% confidence intervals (CI). Haldane’s correction was used when a value was zero in a 2×2 table. The threshold for statistical significance was set at p=0.05, and all tests were two-sided. P-values were corrected according to Bonferroni (pc, corrected p-value) when multiple comparisons were made. Statistical analysis was performed with the SPSS software, version 17 (SPSS Inc., Chicago, IL).

## Results

As indicated in Table 1, 245 subjects were recruited, including 94 VKH patients (median age 40 years; 73.4% female), 39 SO patients (median age 45 years; 43.6% female;) and 112 healthy controls (median age 56 years; 37.5% female;). All patients and controls were Indian Tamils.

**Table 1:** Patient characteristics.

** Variable**	** Value**	**VKH patients (n=94)**		**SO patients (n=39)**		**Control subjects (n=112)**		p-value*
Gender	male (number, %)	25	26.6	22	56.4	70	62.5	<0.001
	female (number, %)	69	73.4	17	43.6	42	37.5	
Age (years)	(median, range)	40	7–86	45	14–73	56	14–79	<0.001
Severity of illness#	moderate (number, %)	20	22.5	14	40.0			0.072
	severe (number, %)	69	77.5	21	60.0			

The asterisk indicates Fisher's exact test for proportions; Kruskal–Wallis test for age. The sharp (hash mark) indicates missing values (n=5 in VKH; n=4 in SO).

We first compared the *HLA-DRB1* intermediate resolution typing results among the three study groups. When considering two-digit allele type frequencies (Table 2), HLA-DRB1*04 displayed a frequency about twofold higher in VKH patients than in controls (20.2% versus 10.3%, OR=2.2, p=0.005, pc=0.067). All other differences between VKH patients and controls were nonsignificant, including the lower frequency of HLA-DRB1*15 in VKH patients (19.1% versus 26.3%, OR=0.66, p=0.10). Comparisons between SO patients and controls did not reveal any significant association.

**Table 2:** *HLA-DRB1* frequencies (generic DR types) in patients with VKH syndrome compared to patients with SO and to healthy controls.

** **	**VKH patient alleles (2n=188)**		**SO patient alleles (2n=78)**		**Control subject alleles (2n=224)**		**VKH versus controls**			**SO versus controls**		
**Allele**	**number**	**%**	**number**	**%**	**number**	**%**	**OR**	**95% CI**	**p-value†**	**OR**	**95% CI**	**p-value†**
HLA-DRB1*01	4	2.1	1	1.3	4	1.8	1.20	0.30–4.85	1	0.71	0.08–6.49	1
HLA-DRB1*03	10	5.3	8	10.3	12	5.4	0.99	0.42–2.35	1	2.02	0.79–5.14	0.18
HLA-DRB1*04	38	20.2	11	14.1	23	10.3	2.21	1.27–3.87	0.005#	1.44	0.66–3.10	0.41
HLA-DRB1*07	30	16.0	7	9.0	40	17.9	0.87	0.52–1.47	0.69	0.45	0.19–1.06	0.07
HLA-DRB1*08	5	2.7	7	9.0	9	4.0	0.65	0.22–1.98	0.59	2.36	0.85–6.56	0.14
HLA-DRB1*09	2	1.1	2	2.6	0	0.0	6.02	0.29–126	0.21	14.7	0.70–309	0.07
HLA-DRB1*10	15	8.0	9	11.5	24	10.7	0.72	0.37–1.42	0.40	1.09	0.48–2.45	0.84
HLA-DRB1*11	6	3.2	2	2.6	5	2.2	1.44	0.43–4.81	0.56	1.15	0.22–6.07	1
HLA-DRB1*12	6	3.2	2	2.6	12	5.4	0.58	0.21–1.58	0.34	0.47	0.10–2.13	0.53
HLA-DRB1*13	16	8.5	3	3.8	21	9.4	0.90	0.46–1.78	0.86	0.39	0.11–1.33	0.15
HLA-DRB1*14	19	10.1	4	5.1	15	6.7	1.57	0.77–3.18	0.22	0.75	0.24–2.34	0.79
HLA-DRB1*15	36	19.1	22	28.2	59	26.3	0.66	0.41–1.06	0.10	1.10	0.62–1.95	0.77
HLA-DRB1*16	1	0.5	0	0.0	0	0.0	3.59	0.15–88.7	0.46	n.a.	n.a.	n.a.

The dagger indicates Fisher's exact test. The sharp (hash mark) indicates Bonferroni correction for multiple comparisons pc=0.067 (n=13). n.a. indicates not applicable.

Similar results were obtained when genotype frequencies were considered (data not shown). HLA-DRB1*04 was present in 37.2% of VKH patients, 25.6% of SO patients, and 19.6% of controls, with a significant difference between VKH patients and controls (OR=2.4, p=0.008, pc=0.094).

**Figure 1:**
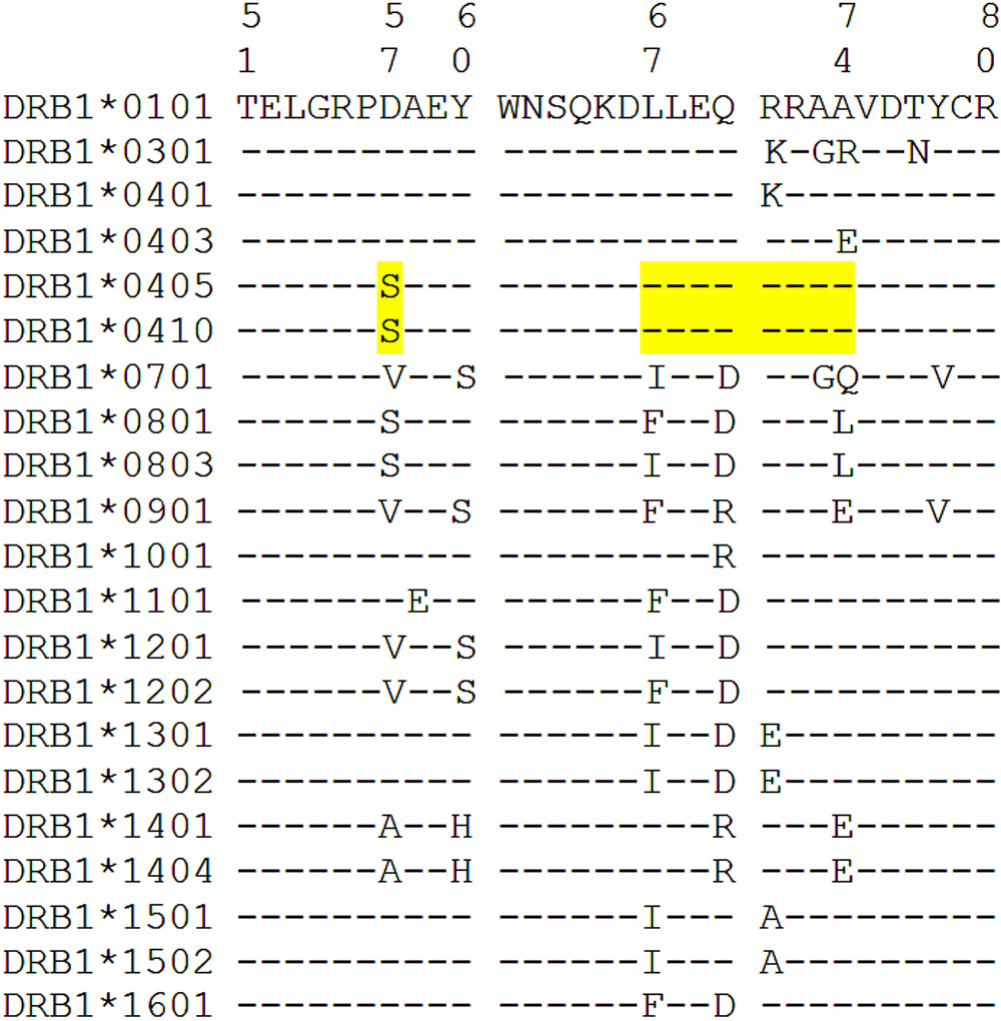
The third hypervariable region sequence of the human leukocyte antigen (*HLA*)*-DRB1* first domain of the alleles identified in patients and controls. Residues 51–80 are represented. In addition to the alleles included in the figure, the following rare alleles were also observed in this study: HLA-DRB1*0305, HLA-DRB1*0407, HLA-DRB1*0804, HLA-DRB1*1106, HLA-DRB1*1111, HLA-DRB1*1415, HLA-DRB1*1506, HLA-DRB1*1507, HLA-DRB1*1509, HLA-DRB1*1514, and HLA-DRB1*1602. Amino acid sequences are shown using the one-letter code. The epitope S57–LLEQRRAA (67–74) is highlighted.

We then considered high-resolution typing for *DR1* and *DR4* alleles (Table 3). Results showed a significantly higher frequency of HLA-DRB1*0405/HLA-DRB1*0410 alleles in VKH patients than in controls (8.5% versus 0.9%, OR=10.3, p<0.001). As illustrated in Figure 1, the HLA-DRB1*0405 and HLA-DRB1*0410 alleles share the S57-LLEQRRAA (67–74) epitope in the α-1 domain of the DR β-chain. VKH patients also showed a slightly, but nonsignificantly, higher frequency of HLA-DRB1*0101 and HLA-DRB1*0403/HLA-DRB1*0407 alleles, which share the LLEQRRA (67–73) epitope with HLA-DRB1*0405/HLA-DRB1*0410, while HLA-DRB1*0403/HLA-DRB1*0407 differ at residue 74 (Ala versus Glu). HLA-DRB1*0405/HLA-DRB1*0410 showed no significant association with SO disease.

**Table 3:** Genotypic frequencies of *HLA-DRB1* alleles within the DR1 and DR4 specificities.

** **	**VKH patient alleles (2n=188)**		**SO patient alleles (2n=78)**		**Control subject alleles (2n=224)**		**VKH versus controls**			**SO versus controls**		
**Allele**	**number**	**%**	**number**	**%**	**number**	**%**	**OR**	**95% CI**	**p-value†**	**OR**	**95% CI**	**p-value†**
HLA-DRB1*0101	4	2.1	1	1.3	4	1.8	1.20	0.30–4.85	1	0.71	0.08–6.49	1
HLA-DRB1*0403	18	9.6	7	9.0	16	7.1	1.38	0.68–2.78	0.38	1.28	0.51–3.24	0.62
HLA-DRB1*0405	10	5.3	1	1.3	1	0.4	12.5	1.59–98.8	0.003#	2.90	0.18–46.9	0.45
HLA-DRB1*0407	1	0.5	0	0.0	0	0.0	3.59	0.15–88.7	0.46	n.a.	n.a.	n.a.
HLA-DRB1*0410	6	3.2	2	2.6	1	0.4	7.35	0.88–61.6	0.051	5.87	0.53–65.6	0.17
HLA-DRB1*0405 or *0410	16	8.5	3	3.8	2	0.9	10.3	2.34–45.5	<0.001	4.44	0.73–27.1	0.11

The dagger indicates Fisher's exact test. The sharp (hash mark) indicates Bonferroni correction for multiple comparisons pc=0.017 (n=5). n.a. indicates not applicable.

Results were similar when genotype frequencies were considered (data not shown). HLA-DRB1*0405/HLA-DRB1*0410 was present in 17.0% of VKH patients, 7.7% of SO patients, and 1.8% of controls, with a significant difference between VKH patients and controls (OR=11.3, p<0.001).

Disease severity could be assigned for 89 VKH patients. The frequency of HLA-DRB1*0405/HLA-DRB1*0410 alleles was slightly higher in more severe disease conditions, but the difference was not significant (9.4% versus 7.5%, p=1).

## Discussion

In the present study, we found a significantly higher frequency of the HLA-DRB1*0405/HLA-DRB1*0410 alleles in a cohort of 94 VKH patients from southern India compared to healthy controls (Table 3). We propose that the epitope S57-LLEQRRAA (67–74) in the third hypervariable region of the DR β-1-chain (Figure 1) could be relevant to the susceptibility to VKH syndrome. In this cohort, residue S57 alone could not account for the susceptibility, as proposed previously [8], since S57-positive HLA-DRB1*08 allele frequency did not significantly differ between VKH patients and controls (Table 2).

Alternatively, if the weak association of VKH disease with HLA-DRB1*0101 and HLA-DRB1*0403/HLA-DRB1*0407 is considered as relevant, the susceptibility epitope shared by the five alleles would be LLEQRRA (67–73) in the third hypervariable region of DRβ1 molecules (Table 3, Figure 1). This epitope exhibits considerable similarity to the rheumatoid arthritis-shared epitope [19]. The observed association of VKH with DR1 in some patient cohorts [13] would support this possibility, although HLA-DRB1*0403/HLA-DRB1*0407 were not increased among 48 Japanese VKH patients [15].

Because the association between VKH and HLA-DRB1*0405/HLA-DRB1*0410 was much stronger than with HLA-DRB1*0101/HLA-DRB1*0403/HLA-DRB1*0405/HLA-DRB1*0407/HLA-DRB1*0410 (OR 10.3 versus 2.4) in the present study, our data favor the hypothesis of a susceptibility epitope shared by HLA-DRB1*0405 and HLA-DRB1*0410 alleles in south Indian patients. The nonpolar alanine at residue 74 of HLA-DRB1*0405/HLA-DRB1*0410 could be crucial in increasing the affinity of antigenic peptide(s) to the HLA-DR4 molecule and/or T cell receptor recognition. A role of Ser at position 57, predicted to point toward the peptide-binding cleft, should also be considered.

In a cohort of 57 Japanese patients, the association of HLA-DRB1*0405/HLA-DRB1*0410 was stronger with prolonged than nonprolonged VKH disease [16]. Our results similarly showed a slightly higher frequency of HLA-DRB1*0405/HLA-DRB1*0410 in patients with severe compared to moderate disease conditions, but the difference was not statistically significant. A role of DQB1*0402, which is in linkage disequilibrium with HLA-DRB1*0405/HLA-DRB1*0410, seems unlikely because 40% of 15 tested VKH patients were DQB1*0402 negative (data not shown).

The HLA-DR susceptibility epitope could slightly differ in populations with different genetic backgrounds. Accordingly, south Indian VKH patients in the present study might be closer to other Asian cohorts than to Hispanics. However, in a recent study of North Indian VKH patients, no association with DR4 was observed, which is possibly related to the high prevalence of patients with incomplete syndrome and to the low frequency of HLA-DRB1*0405 in this population [20,21]. When compared with a north Indian population [20], the present south Indian control population was characterized by a twofold higher relative frequency of HLA-DRB1*0403 (69.6% versus 34.8% in the north Indian population), whereas frequencies of HLA-DRB1*0405 and HLA-DRB1*0410 were lower (4.3% versus 11.2%) and comparable (4.3% versus 3.4%), respectively.

Even though the present results reveal a strong association of VKH with the HLA-DRB1*0405/HLA-DRB1*0410 alleles, the frequency of these two alleles was relatively low in this south Indian population. In conclusion, although highly significant, the immunogenetic basis of HLA-DRB1 associations with susceptibility to VKH syndrome seems to be weaker in south Indian Tamils than in Japanese or Hispanic patients. Additional genetic systems, such as killer immunoglobulin-like receptor–HLA interactions could also play a role, as suggested recently in a small cohort of Mestizo VKH patients [22]. Finally, no *HLA-DRB1* association could be observed with SO, which is in accordance with the clearly different physiopathology of both diseases.
